# Microbiome Analysis via OTU and ASV-Based Pipelines—A Comparative Interpretation of Ecological Data in WWTP Systems

**DOI:** 10.3390/bioengineering9040146

**Published:** 2022-03-29

**Authors:** Jan Torsten Jeske, Claudia Gallert

**Affiliations:** Faculty of Technology, Microbiology-Biotechnology, University of Applied Science Emden/Leer, 26723 Emden, Germany; claudia.gallert@hs-emden-leer.de

**Keywords:** OTU, ASV, Illumina, pipelines, bioinformatics, wastewater treatment, microbiome, sequence processing, sequence data handling, microbial community composition

## Abstract

Linking community composition and ecosystem function via the cultivation-independent analysis of marker genes, e.g., the 16S rRNA gene, is a staple of microbial ecology and dependent disciplines. The certainty of results, independent of the bioinformatic handling, is imperative for any advances made within the field. In this work, thermophilic anaerobic co-digestion experimental data, together with primary and waste-activated sludge prokaryotic community data, were analyzed with two pipelines that apply different principles when dealing with technical, sequencing, and PCR biases. One pipeline (VSEARCH) employs clustering methods, generating individual operational taxonomic units (OTUs), while the other (DADA2) is based on sequencing error correction algorithms and generates exact amplicon sequence variants (ASVs). The outcomes of both pipelines were compared within the framework of ecological-driven data analysis. Both pipelines provided comparable results that would generally allow for the same interpretations. Yet, the two approaches also delivered community compositions that differed between 6.75% and 10.81% between pipelines. Inconsistencies were also observed linked to biologically driven variability in the samples, which affected the two pipelines differently. These pipeline-dependent differences in taxonomic assignment could lead to different conclusions and interfere with any downstream analysis made for such mis- or not-identified species, e.g., network analysis or predictions of their respective ecosystem service.

## 1. Introduction

The most important tool in a microbiologist’s toolbox for unraveling the composition, and per proxy functioning, of microbial ecosystems today is the analysis of the 16S ribosomal RNA gene via next-generation sequencing (NGS) methods. As only a small fraction of the microbiome is accessible for cultivation, cultivation-independent methods are obligatory to identify the full range of organisms involved in various microbe-driven ecosystem services [1,2,3]. Using the 16S/18S ribosomal RNA gene as a phylogenetic marker has, with ongoing validation and improvement [4,5,6,7,8], been at the center of such efforts over the past decades. Its suitability for this purpose lies in its conserved function, omnipresence in all three domains of life, and the contained variable and conserved regions. Major drawbacks of NGS methods are typically short fragment lengths and sequencing-technology-related technical artifacts, which often accumulate toward the end of a read [9,10,11]. Such problems may often be mitigated by the choice of region and increased overlap. Yet, doing so further reduces the output fragment length, and consequently decreases taxonomic resolution. NGS methods, in a historical compromise to those technological restrictions [4], typically only look at small but highly variable regions of the 16S rRNA gene to assess taxonomic relationships. Among those, the V3–V4 region is probably the most frequently used [12,13,14,15]. However, the above-mentioned drawbacks fall far short of the benefits that NGS methods provide; they still need to be addressed appropriately. In the past, this has been carried out by quality filtering and averaging them out via sequence-clustering at a similarity threshold [16,17,18]. The resulting consensus sequences are referred to as operational taxonomic units (OTUs). Said threshold is typically set at 97% identity, with the OTU representing a consensus sequence at the respective centroid of the cluster. The inherent problem is that these OTUs are internally generated and analysis-specific. Thus, there is no direct comparability given with other studies, and each comparison has to be made indirectly via cross-referencing with databases such as Greengenes or SILVA [5,19]. This, in turn, has to be performed under the assumption that both OTUs, i.e., both 97% sequence similarity threshold centroids, accurately represent the organism present in the respective sample. This typically limits comparisons to be made at the *Genus* level at best.

Recent alternative analysis pathways seek to incorporate sequencing error profiles in order to compute algorithms that correct these technical artifacts and revert them into exact amplicon sequence variants (ASVs). These claim to be accurate up to a resolution of single-base transitions and, instead of short-hands (OTU), generate actual sequences (ASV) that are directly comparable across studies [20,21]. Their contra-point is a limited capability to deal with small or missing overlap regions, and an inability to deal with undefined bases (i.e., N’s), which is less of a problem for the OTU approach. Additionally, ASVs are also less widely accepted and used, which may partially be linked to them being less easily incorporated into text, given the name may be as long as a 300 bp sequence instead of a three-letter word and a three-digit number [22,23]. However, simplifying and using a short-hand for an ASV would somehow equally distort its original intent, while it may still be necessary for publication purposes. On the plus side, such newer approaches also come with a greatly reduced need for computation power and a much-improved ease of access compared with the more hands-on and individualized pipelines. However, this might also be a slightly double-edged blade, as it includes a potential for user- and input-defined biases or errors to be more frequent, and they potentially go unnoticed altogether.

Additional recent work has shown that individual pipelines themselves may be biased toward certain phyla [15,21]. This stands in harsh contrast to the attempted certainty of results, which is inadmissible for comparisons and accurate predictions of the function of microbial communities, and their respective individual members. Thus, there is a growing need to no longer assess sequencing data in isolation, but instead more often test and re-evaluate the common ground. This aims to close the gaps and uncertainties that arise between the available pathways of data-handling in order to more accurately, and with more certainty, explore the underlying biological facts.

The same is also true with an eye on current advances in technology such as nanopore sequencing [24], which could allow a return to the use of the entire 16S *rRNA* gene for taxonomic assignments, without sacrificing throughput quantity or speed. However, this might come at the cost of a slightly lowered average sequence quality, which might present a new challenge, especially for the more advanced pipelines [4].

In order to address the pros and cons of both options for sequence data analysis, this work aims to raise awareness for some of the aforementioned challenges, with special emphasis on implications for wastewater treatment data, where sequencing methods are becoming more frequent for assessing and even monitoring functionality via community composition and amplicon sequencing [25,26,27]. However, the description of methods and interpretation of results are also applicable to other fields of research, and different habitats and microbiomes. To elucidate some of the underlying challenges, within the framework of research outside of a proof-of-principles scenario, lab-scale anaerobic co-digestion (Co-AD) of primary and waste-activated sewage sludge (PWASS) together with food and kitchen waste (FW) are used as a mostly well understood, yet still current, field of research, simulating wastewater treatment processes that are linked to various disciplines ranging from microbiology to energies [28,29,30].

## 2. Materials and Methods

### 2.1. Sampling and Experimental Setup for Simulation of Anaerobic Co-Digestion Co-AD 

Six lab-scale reactors, 1 L total reactor volume, each with 500 mL of continuously stirred working volume, were operated over a period of 170 days, during which the temperature was kept constant at 55 °C. Inoculum was obtained from a thermophilic anaerobic digester located at a midsized wastewater treatment plant (WWTP) in northern Germany on 11 January 2021. For daily feeding, a mixture (PWASS) of primary sludge (PS, ~40%) and waste-activated sewage sludge (WAS, ~60%) was collected from a pre-thickener, directly upstream of an AD reactor at another midsized WWTP in northern Germany, approximately every 4 weeks. PWASS was mushed, distributed in daily portions, and stored frozen before use. For feeding, 50 mL of reactor content of each control reactor (n = 3) was replaced with 50 mL of temperature-equilibrated PWASS every 24 h, excluding weekends, leading to a hydraulic retention time (HRT)-stabilized system with an HRT of 14 days. Experimental reactors (n = 3) were treated equally, but, following a one-month feeding period without co-substrate addition (T_1_ = 0%), were supplemented with food waste (FW) as a locally available and easy degradable co-substrate. The added amount of the co-substrate was calculated as a percentage of the volatile solids (VS) of the respective PWASS-batch and increased stepwise in 10% increments over the duration of the experiment from 0% to 40%. Food and kitchen wastes were obtained from a Chinese restaurant where they were collected over one day, after which they were mushed and stored at −20 °C until use. The chemical composition of the co-substrate and example of the PWASS can be seen in [31].

### 2.2. Analytical Procedures

Reactor performance was controlled via online-monitoring methane and biogas production using methane sensors (BlueSens, Herden, Germany) and MilliGascounter® (Ritter, Bochum, Germany). Volatile Fatty Acids (VFAs) were measured weekly via gas chromatography according to [32], and pH was measured offline in the reactor excess daily during feeding (Hanna Instruments, Vöhringen, Germany). Total (TS) and volatile solids (VS) were measured by gravimetric analysis according to the Standard Methods 2540B and 2540E, respectively [33].

### 2.3. DNA Extraction and 16S rRNA Gene Amplicon Sequencing 

Samples for DNA extraction were taken regularly from the lab-scale reactors and stored at −20 °C. Samples at the end of each co-substrate addition period (n_T_ = 5) from each reactor (n_R_ = 6) were used together with the PWASS (n_P_ = 5) and the inoculum sample for DNA extraction, resulting in a total of 36 individual samples. DNA for amplicon sequencing was extracted using the Soil DNA Isolation Plus Kit (Norgen, Thorold, ON, Canada) in triplicates according to the manufacturer’s instructions. DNA concentrations ranged between 10.7 ng ${\mu L}^{-1}$ and 338.6 ng ${\mu L}^{-1}$ with an average of 50.9 ng ${\mu L}^{-1}$. Extractions yielding less than 10 ng ${\mu L}^{-1}$ were repeated until sufficient DNA (quality and quantity) was obtained. The hypervariable V3–V4 region of the 16S rRNA gene was used as marker to analyze the microbial community composition in the thermophilic lab-scale reactors and the “psychrophilic” PWASS (mixture of primary and excess sludge from the activated sludge system operated under ambient conditions). Sequencing of barcoded amplicons was performed using the Illumina MiSeq platform, with V2 chemistry (Illumina Inc., San Diego, CA, USA) according to the manufacturer’s instructions. Primers Pro341f/Pro805r [14] targeting both the domain *Archaea* and *Bacteria*, respectively, were used to generate barcoded amplicons for sequencing. Libraries were prepared using the NexteraXT kit (Illumina Inc., San Diego, CA, USA).

### 2.4. Sequence Analysis OTU 

Sequence processing was performed according to the method of Dyksma et al. [34] In summary, raw reads were filtered and low-quality reads removed. Paired end reads were then assembled, trimmed, denoised, had chimeras removed, and aligned to SILVA database v138.1. Sequences were then clustered into operational taxonomic units (OTUs) at 97% similarity level with VSEARCH [16] and classified using the SINA-classifier. After quality filtration, a total of 18,397,934 reads were obtained from 36 samples. For Cutadapt, a minimum adapter overlap threshold for the trimming of 6 bases was used, with an adapter error rate of 0.1. BBMerge was set to default merging strictness with a quality trim threshold set to 10. SINA classification was performed with a minimum length of 100 bp with a quality score filter of 20 applied to the 3′ end for filtering, and alignment was performed based on the SILVA REF99 SSU reference database version 138.1.

### 2.5. Sequence Analysis ASV 

Analysis of the sequencing data using the ASV-based pipeline was performed using the DADA2 pipeline, with DADA2 v1.18.0 and R version 4.1.0 (2021-05-18)—"Camp Pontanezen." Sequences were processed according to the tutorial instructions for the A DADA2 workflow for Big Data: Paired-End (1.4 or later) [20,35], with the following adjustments made. Sequences were trimmed left 17 bases to remove residual primers, truncQ was lowered to 2, and the option to truncLen was removed. For chimera removal, the minFoldParentOverAbundance threshold was increased to 8. Taxonomy assignment was performed based on the SILVA REF99 SSU reference database version 138.1.

### 2.6. Data Handling

Sequence analysis was performed with R [36] version 4.1.2 (2021-11-01)—"Bird Hippie" using the packages phyloseq [37], vegan [38,39], and microbiome [40]. OTU data were used unrarefied and rarefied by the minimum number of reads (41,635) with vegan, and subsequently converted to a phyloseq object. Only rarified data were used for final community analysis. Eukaryotic, mitochondrial, and chloroplast sequences were removed, as well as OTUs that remained unclassified on the domain taxonomic level. Equally, an ASV phyloseq was created according to the tutorial described in 2.5. Both phyloseq objects were then merged. The resulting phyloseq was rarefied using the rarefy_even_depth function by minimum sample_sums, as included in the phyloseq package, and thereafter condensed at the *Genus* level by taxa agglomeration for further processing and combined analysis. Alpha diversity was analyzed using vegan and microbiome. The relative abundance of the different taxons was determined using the transform “compositional” function included in the microbiome package, after normalization via rarefying. Permanova analysis was performed using the adonis2 function implemented in vegan. NMDS ordination was performed based on the bray-curtis dissimilarity on scaled reads using functions implemented in the vegan and phyloseq packages, and graphical presentations were made using the packages base, ggplot2 [41] and cowplot [42]. For statistical analysis, PWASS, control, and experimental reactors were regarded as three distinct levels of the factor treatment. Similarly, temperature was considered a factor with two levels, either thermophilic or psychrophilic, and pipeline was considered as a factor, also with two levels (OTU/ASV). Timepoint, i.e., the experiment runtime, was treated as a continuous time variable.

## 3. Results

### 3.1. Operational Data and Reactor Performance

In order to show the capacity of a WWTP with a thermophilic anaerobic digestor for producing additional biogas when feeding available and easily degradable substrates such as food waste, semicontinuous operated lab-scale reactors were operated with the same HRT (days) and OLR (g VS m^3^ d^−1^) as the full-scale WWTP. Food waste as a co-substrate was added as a percentage of the organic loading rate OLR of the main substrate PWASS. The average daily biogas production and methane content of both control and experimental reactors of each phase (0–40% co-substrate) are summarized in Table A1. With increasing amounts of co-substrate, the total amount of biogas and methane increased, and the control and experimental reactors ran stable for the whole operation period without any process disturbances.

### 3.2. Sequencing Pipeline Data Overview 

The Illumina sequencing output reported an average quality of Q30 ≥ 81.9%. Using Cutadapt, the OTU-based pipeline converted the raw reads into 18,397,934 total reads from forward and reverse reads, with 9,198,967 total reads each. These represent 3,695,630 and 3,061,507 unique sequences from forward and reverse reads, respectively, from which BBmerge, dereplication, and chimera removal using VSEARCH [16] produced a total of 7,643,311 remaining reads (≈82.4%), with 73,003 unique sequences after clustering at a 97% identity threshold. Those were then converted to a phyloseq database for further processing, both with and without rarefying [43,44]. The ASV-based pipeline combines all of the above-stated steps but also includes a quality-based error correction mode instead of the 97% clustering inherent in the OTU-based approach. Using this error correction and manual, size, and base-position-based trimming and merging of the samples, the DADA2 [20] pipeline produced a total of 13,342,124 reads with 2,327,901 unique sequences. The error algorithm that is hardcoded in the pipeline uses a user-defined minimum of bases to calculate the error rates and reads in bases successively from one sample after another, until it reaches a solution. In this case, the standard number of bases suggested was used, resulting in 126,004,658 total bases in 539,888 reads from 3 samples for the forward reads, and 100,116,913 total bases in 427,973 reads from 2 samples from the reverse reads being used to calculate the error rates. Furthermore, an abundance threshold of 0.001% was applied to the ASV and OTU datasets to eliminate rare taxa. Parallel OTU and ASV datasets were agglomerated at the *Genus* level to be processed and compared within one dataset. The resulting number of taxa for each approach can be seen in Table 1.

### 3.3. α-Diversity Comparison of Pipeline Outcomes

**Figure 1:**
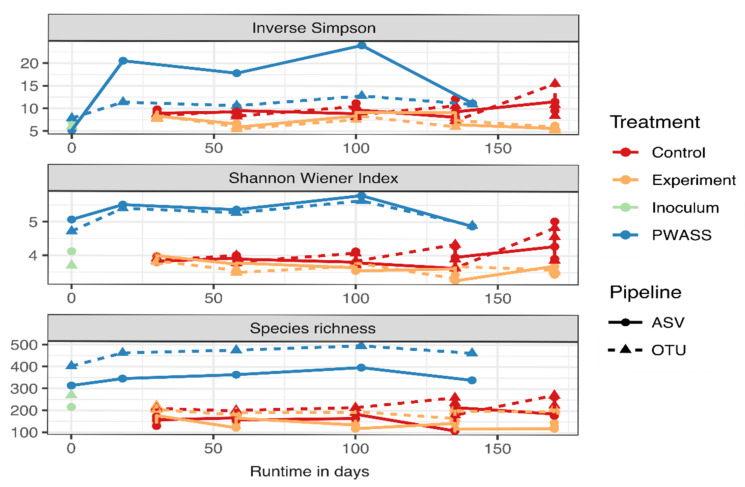
Comparison of the two pipelines for observed *α*-diversity over time, expressed via a set of different *α*-diversity indices. Sample types, i.e., treatments, are represented with color-coding, while pipelines are represented by differing symbols, and dashed and solid lines.

Based on the full, but rarefied, dataset, α-diversity indices were calculated including the Shannon Wiener index, richness, and evenness estimates (Figure 1). Largely stable communities were observed over time in the lab-scale reactors, with only little changes over time in both experimental and control reactors. Trends for diversity development in the anaerobic digester/experimental reactor community were similar across time and diversity index between the two pipelines, although the OTU-based pipeline data typically reported slightly higher diversity than what was observed for the ASV pipeline. However, differences were observed for the PWASS microbial communities, where, while some diversity indices (e.g., Shannon Wiener index) reported similar values, other diversity estimators strongly differed between the two pipelines (Figure 1). In the PWASS, and in opposition to the experimental reactors, very different behavior, up to a partial trend reversal, was observed for some estimators—especially, but not limited to, evenness indices. The inverse Simpson index is especially notable, for which OTU und ASV indices for the AD communities were almost identical, while the PWASS differed at times by a factor greater two.

### 3.4. β-Diversity Comparison of Pipeline Outcomes—Prokaryotic Community Composition on Phylum Level

**Figure 2:**
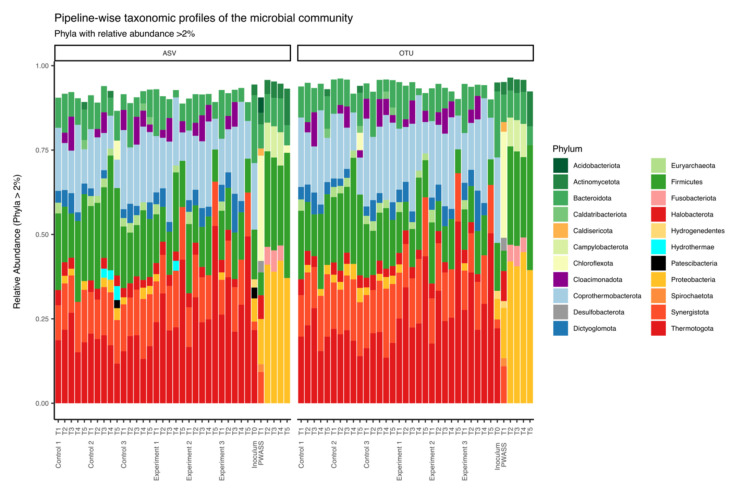
Taxonomic profiles of the microbial populations observed by the two pipelines presented as bar charts. For better visibility, only those phyla that represent more than 2% of the total community are included in the bar-chart. Microbial community composition observed by the ASV-pipeline is shown on the left; those observed in the OTU-based pipeline are shown on the right. Bars from left to right of each plot represent: control samples over five individual timepoints spanning 170 days in biological triplicates, experimental reactors over the same five individual timepoints, with 10% increment increases in co-substrate addition from 0% (T_1_) to 40 % (T_5_), also in biological triplicates, and inoculum (T_0_) and PWASS samples correlating to the five experimental timepoints.

The *Phylum* level composition was overall in good agreement between both pipelines, for the 36 samples and the 4 respective treatments (control, experiment, inoculum, and PWASS) with individual variations between samples and treatments being identified by both pipelines, mostly to similar degrees (Figure 2). The bacterial community in the experimental and control reactors consisted of decreasing relative abundance, primarily of members of the *Phylum Thermotogota*, followed by *Coprothermobacterota*, *Synergistota*, *Firmicutes*, and *Bacteroidota*, with members of the phyla *Dictyoglomota*, *Clocacimonadota*, *Caldatribacteriota*, and *Proteobacteria* contributing in the lower single-digit percentages (Figure 2). The archaeal community contributed 5% of the total relative abundance, as identified almost identically by both pipelines. Of those, 59% were identified as members of the *Halobacterota*, primarily *Methanothrix*, and 40% and 41% in the control and experimental reactors, respectively, being assigned as members of the *Euryarchaeota* (*Methanothermobacter*). A similar composition was observed in the inoculum, although there, both pipelines detected *Archaea*, *Dictyoglomota*, and *Cloacimonadota* with 0.9%, and *Synergistota* with 3% only, with an increased relative abundance of *Coprothermobacterota* and even more pronounced *Bacteroidota* (see Figure 2 and Table S1 in Supplementary Materials for a comprehensive list). The PWASS microbial community differed considerably from the above-described microbial assemblages, with more pronounced variability between samples, i.e., timepoints, than what was observed for the more stable AD laboratory reactors. 

The between-pipeline variability was also much greater in the PWASS than in the other treatments. Both pipelines reported an overall similar microbial community composition, typical for PWASS. As such, a majority of the microbial community was made up of *Proteobacteria* and *Firmicutes* (Figure 2), together already contributing 63% of the total microbial community, although with individual contributions differing. In decreasing relative abundances, *Bacteroidota*, *Chloroflexota*, *Actinomycetota*, *Campylobacterota*, *Fusobacteriota*, and *Synergistota*, as well as some *Archaea* belonging to the phylum *Halobacterota*, all in the single-digit range, made up the majority of the remaining microbial community. Among those, only *Halobacterota* and *Fusobacterota* (Figure 2) were reported in equal numbers by both pipelines. All other phyla differed between 1% and 4%. This encompassed ranges between 34% and 36%, i.e., a difference of ~2% total community composition, and ~5% relative to one another for *Proteobacteria*, to 1.2% and 0.3% for *Acidobacteriota*, equaling ~1% of the total community, but deviating by 400% between the two pipelines (Table 2). Pipeline-based deviations in abundances on the *Phylum* level that are either greater than 1% total community composition or deviate by more than 20% between pipelines (times fold change equal to a factor 1.2), while, at the same time, contributing at least 0.5% to the total community composition in at least one of the pipelines can be seen in Table 2, with a comprehensive list of *Phylum*-level relative abundances in Table S1. In sum-total, the community composition differed between 6.75% and 10.81% (sum ∆ of total community per treatment—PWASS, inoculum, control, experiment, Table S1 in Supplementary Materials) on *Phylum*-level composition based purely on the used pipeline.

### 3.5. 𝛽-Diversity Comparison of Pipeline Outcomes—Prokaryotic Community Composition on Genus Level

**Figure 3:**
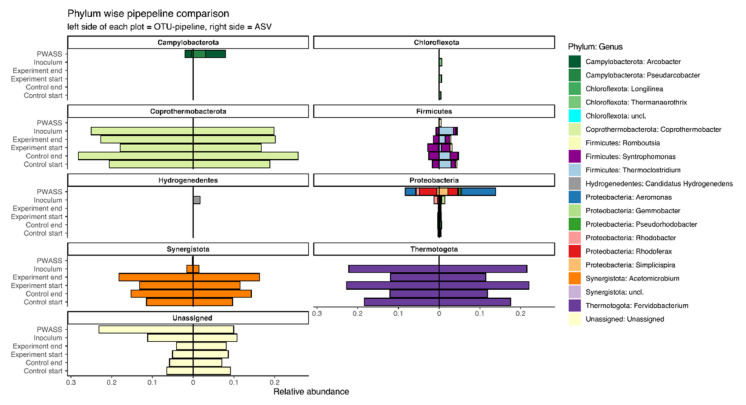
Divergence bar plot showing samples from the inoculum, representative lab-scale reactors, and the PWASS. Plotted are the taxa agglomerated at *Genus* level with the top 20 Permanova coefficients identified as a response to the pipeline as a categorical factor. Bars are plotted *Phylum*-wise, with bars extending from the centerline to the left representing the relative abundance of the respective taxon in the VSEARCH-based OTU pipeline, and bars extending to the right representing the ASV-generating DADA2-pipeline.

While often being consistent between the two pipelines in general, differences that already became apparent on the *Phylum* level subsequently also manifested on the *Genus* level. As such, the general *Genus* representation in the AD reactor samples were largely similar, with, however, a few selected taxa differing significantly between the two pipelines (Figure 3). Especially, genera belonging to the *Firmicutes*, *Chloroflexota*, *Patescibacteria*, and *Hydrothermae* were affected, especially with *Chloroflexota* and *Firmicutes* contributing significantly to pipeline-based differences, and with several genera not being picked up by one or the other pipeline (Table 2, Figure 3). As observed with the α-diversity indices, differences between the pipelines were more prominent in the PWASS sludge samples. Especially, genera belonging to the *Campylobacterota* and the *Proteobacteria* were assigned with significantly different abundances, differing by as much as 3% of the total microbial community for *Aeromonas*, or by 400% between the pipelines (4% ASV, 1% OTU), respectively (Figure 3).

### 3.6. Statistical Analysis of Pipeline Outcomes in the Light of Ecological Data

**Figure 4:**
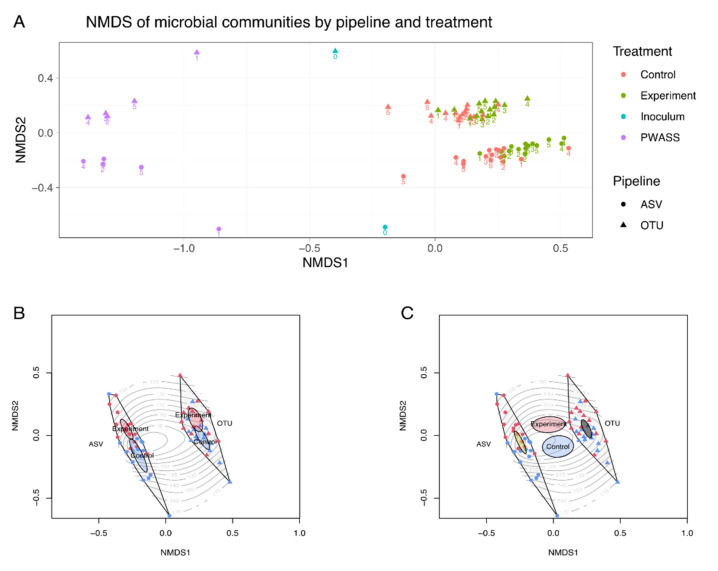
Nonmetric multidimensional scaling plot of the microbial populations identified by the two pipelines. (**A**) Shown is the full set of samples including PWASS, inoculum and control, and experimental reactor samples for both pipelines. Treatment levels, i.e., sample origin, is depicted by colors, with numbers representing the sampling time-point between 1 and 5 (0 for inoculum). The two pipelines are represented by different shapes. (**B**) and (**C**) Nonmetric multidimensional scaling ordination of the experimental and control thermophilic anaerobic digestion reactors only. Shape represents the pipeline, with triangles representing the OTU and circles representing the ASV pipeline. Added to the ordination is experimental runtime as contour lines, and pipelines are additionally depicted by polygons encompassing all samples belonging to either one or the other pipeline. 95% confidence intervals are included in the ordination as ellipsoids (**red** = experimental reactors, **blue** = control reactors); (**B**) 95% confidence intervals are calculated and plotted pipeline wise; (**C**) 95% confidence intervals are calculated for the whole set of samples, with addition of confidence intervals for the two pipelines in yellow (ASV) and grey (OTU).

Several models were tested via Permanova analysis to identify correlations between the observed community composition and the different sample types and pipelines—only including the thermophilic AD reactors; with AD-reactors and PWASS combined; and both sets either as a single combined dataset or with the samples being nested in the respective pipeline. Significant differences in the individual factors, as well as interacting effects, can be seen in Table 3, and in the corresponding NMDS analysis (Figure 4A). With inclusion of the PWASS samples in the analysis, all factors except the treatment (PWASS was weighed in as a treatment level) were found to be less significantly correlated. Simultaneously, when the design was crossed, i.e., the factors were nested within the respective pipeline, the null-hypothesis could no longer be rejected, in the AD-only dataset and the dataset including the PWASS as well. The latter, however, did show significant results when temperature was included as an interaction factor together with the treatments. Corresponding NMDS analysis of the full dataset revealed six separate clusters (Figure 4A). These were found to be two for the PWASS, one for each pipeline, and another set of two for each pipeline, for the experimental and control AD reactors, respectively. To better resolve pipeline-derived differences, the NMDS was also repeated on the reduced dataset only including the AD-Reactors (Figure 4B,C), and again, two larger clusters were found, representing the pipelines, that were internally divided by the respective treatment levels along a runtime—i.e., timepoint—gradient.

## 4. Discussion

### 4.1. Minimizing Sequencing Data Bias during Microbial Community Analysis 

The presented results outline several challenges for methods handling sequencing data bias, which originate in the use of different approaches, which attempt to overcome sequencing and PCR bias in community analysis [10,11,20,45]. On a broader scale, this work also looks at the implications different handling approaches might have on the interpretation of data in microbial ecology. This is exemplified on a WWTP experimental setup. Established systems of sequence and data handling, and workflows are constantly changing, while the amount of data available, and in parts also the computation power to deal with them, is also increasing exponentially. The increase in options of workflows, pipelines, and data analysis pathways brings more choice, but also more doubt into the reliability of current and previous data. The goal in going forward thus has to be to optimize data handling in order to consistently and reliably improve the comparability between studies, despite the approaches of how to get there becoming more and more diversified. Switching from de novo operational taxonomic units (OTUs) to amplicon sequencing variants (ASVs) has the potential to achieve these goals and allow for such required direct comparisons across studies, especially in the backlight of the current ongoing revision of nomenclature in prokaryotes [46]. Consequently, it is imperative that the quality of analysis and data for each workflow ultimately meets even higher standards than OTU-derived data have achieved so far. This also means that the algorithms that are being used to produce these data are constantly improved upon and checked for their validity, applicability, and robustness. As for now, two main operational frameworks are at play on how to deal with sequencing biases.

These are OTU-generating approaches based on clustering of sequences (e.g., USEARCH-UPARSE, VSEARCH) [16,18], k-mer-based strategies basically averaging out sequencing errors, and ASV-generating algorithms based on inferred error frequency to correct and revert sequencing errors (e.g., DADA2, Qiime2, UNOISE3) [17,20,45]. While OTUs have been used for a long time, and data and experience on them are plentiful, recent work has repeatedly proven the robustness and reproducibility of ASV-generating algorithms in mock communities [17,47], or in community data from environmental studies, often centered around the human microbiome or human microbiome-associated environments [48,49]. One or both of these approaches were applied to a wastewater treatment setting combined with experimental manipulation, and with samples from thermophilic and psychrophilic environments. The ease of use that some pipelines provide unfortunately also increases the probability of user-derived errors, which, due to the ease of use, have an additional potential to go unnoticed more readily. In general, the comparability between pipelines is excellent [21,47,48,50]. However, as this work outlines, some particular prerequisites should be met, in sequencing and sequence handling design that otherwise may affect pipelines differently, and thus will potentially lead to pipeline-derived differences in the interpretation of what factually is identical data.

### 4.2. Equalizing Sequencing Data Bias during Microbial Community Analysis

Previous research has repeatedly reported that clustering and OTU-based approaches such as that used here, VSEARCH, but similar for USEARCH-UPARSE, MG-RAST, or QIIME and others [16,48,51,52], have the frequent problem of overestimating diversity and producing spurious amounts of OTUs, especially including singletons and doubletons. The results presented in this work are no exception to this; however, problems arising from such spurious OTUs are easily mitigated via relative abundance thresholds (Table 1). Reitmeier et al. [51] proposed abundance cutoffs as strict as 0.25% relative abundance to be used as a threshold for OTU-based analysis. For data in this work, a cutoff as little as 0.001% was sufficient to remove enough spurious or rare taxa to be within the ranges produced by the ASV pipeline used (Table 1). The ASV pipeline itself is specifically designed to output fewer to no spurious sequence variants at all, while also retaining the precision to identify single-base difference variants [20]. A measure as small as that, combined with rarefication [43,44,53], already led to good agreement of the resulting data, and thus to the interpretation of alpha diversity data for the experimental and control reactors (Figure 1). Both pipelines, to similar degrees, allow for the same interpretation of the observed alpha diversity indices for the thermophilic laboratory reactors. Both pipelines picked up to a similar degree on the slightly increased diversity in the control reactors when compared to the, with runtime increasing, co-substrate-fed experimental reactors. In contrast, diversity indices diverged between pipelines for the psychrophilic PWASS samples. Alpha diversity in the PWASS samples was expected to show different characteristics overall in comparison with the AD reactors [31]. Differences were expected on a basal level, readily explained by the vastly different habitats. Differences were also expected over time, given the exposure of the PWASS to seasonal, especially temperature, variations [54,55], which is a nonfactor in the temperature-controlled experimental reactors. Following the good agreement of the above-described thermophilic AD reactors, it was also expected that both pipelines would show similar levels of agreement between-pipelines in the PWASS. Yet, several quite drastic differences were observed, which can only be retraced to differences in how the data are being handled by the pipelines. This raises questions concerning the reliability of at least one of those pipelines, when such strongly differing habitats are investigated simultaneously, which is not uncommon in environmental studies.

### 4.3. Complex Relationships: Dissimilar or Similar

In this work, the prominent factor, which apparently affected pipeline-dependent community composition, aside from inherent pipeline biases [15,47,48], was temperature, but any range of strongly differing environmental gradients may lead to similar sequencing analysis, or rather, algorithm-derived biases. NMDS analysis at first glance revealed a similar, clear pipeline dependency of the sequencing outcome. Both pipelines paint a comparable picture concerning the separation of the PWASS from the AD reactors, as well as the internal separation between the AD-reactor treatments. At the same time, both pipelines are equally clearly separated from one another. Alpha-diversity indices had already revealed pipeline-dependent differences in the PWASS samples, so these were removed from the dataset to investigate whether pipelines would lead to different conclusions, when comparing samples from habitats that are more closely related but differ based on experimental treatment levels. NMDS and Permanova of this reduced dataset identified that the choice of pipeline significantly influences the reported community composition, as does the treatment applied to the reactors (Table 3). Additionally, NMDS also revealed that differences between the pipelines are in fact greater than those observed in the divergence of the treatments over time and with increasing co-substrate addition. This becomes especially apparent in Figure 4B,C with the added 95% confidence intervals included in the illustration for either treatment levels (experiment and control)—either being displayed nested within the pipeline (B) or for the two datasets combined (C). Both pipelines were able to identify a drift in community composition along a time axis, which is co-correlated with the increasing substrate addition. The ASV-Pipeline especially did this in a clearer fashion, suggesting a stronger influence of time and co-substrate than the OTU-based pipeline did. However, similar earlier experiments, with mesophilic reactor conditions, would suggest a less strong influence on the community [25,31]. Strikingly, NMDS and confidence intervals also suggest that even over a duration of more than 170 days, dissimilarities in community composition between pipelines are factually greater than dissimilarities between the microbial populations in the experimental treatments. So far, this would have limited implications on the initial interpretation of the data in isolation; yet, it brings doubt to comparisons made across other studies of a similar scale and focus, if the choice of pipeline differs between studies. With sequencing methods becoming more frequent in direct monitoring of WWTP functioning [26,27], a good level of comparability is imperative even on small scales. While AD reactors typically harbor unique but similar microbial populations [56,57,58], differences on the exact scale observed to differ in this study could very likely be the important common denominator, or dissimilarity, that needs to be identified reliably for cross-study comparisons, including networking and functional approaches. It has to be noted that these results are exemplified with WWTP data but are likely not limited in their applicability to other systems.

Permanova analysis revealed another dividing factor between the two pipelines, which, in parts, had already been identified in the analysis of the alpha diversity. On the whole dataset, the pipeline was found to not be a significant factor correlating with community composition. However, as soon as an interaction of the pipeline with treatment, thus addressing the strong biologically backed differences between the PWASS and the AD reactors, was included in the analysis, the pipeline was found to significantly correlate with the community composition. In theory, this should not affect the outcome, if both pipelines would give the same, or at least highly similar, results. Permanova, furthermore, identified several significant factors correlating with the community composition such as co-substrate addition and concentration, i.e., runtime of the experiment, or combinations of these factors. All of these are known and likely to impact community structure from a perspective of biological reasoning, which is also represented in their respective statistical significance. Yet, once pipeline was included in the analysis, meaning the treatments, temperature, and timepoint individually were analyzed in respect of—i.e., in an interaction with—the pipeline (Table 3), the combination of those factors with the pipeline would no longer return a statistically significant result, unlike that in isolation. This means the differences between the pipelines were large enough that the individual null-hypothesis concerning a non-normal distribution of the microbial populations as a response to the changes in their environment could no longer be rejected. On the reduced dataset, this would lead to the conclusion that the choice of pipeline would affect the community composition more than any of the actual treatments or environmental factors would, which has no foundation in experimental reality.

One peculiar observation was made in the full dataset, as once temperature was also included as a co-correlating factor, thus dividing the experimental reactors and the PWASS in another dimension, a statistical significance of the combined effects, nested in the pipeline, could be detected (Table 3). The ASV algorithm used here calculates error rates to correct for sequencing errors on a subset of samples, which are then applied to each sample of the entire run, individually. This means that error rates, which are unknowingly trained and calculated on a sample possibly exclusive from a thermophilic environment, are then applied to correct sequencing data, and base transition rates, from a psychrophilic environment. In opposition, the k-mer-based clustering strategy that is used in the OTU-based approach is performed on the entire dataset at once, with the downside of being extremely demanding in computation power, but not affected by such random factors, i.e., data that are used to train the algorithm having to also accurately represent base transition rates of the entire dataset. The latter, however, is a statistical fallacy that is in contradiction with the underlying biology and rarely in agreement with the study design, when samples that are analyzed together in one run, coming from different extremes and temperate habitats at the same time, encompass different codon usage for functional genes and metagenomic studies [59], GC content [60,61], and eukaryotic and prokaryotic sequences simultaneously in one sample, but only prokaryotic in another, etc. A difference in the treatment of extreme environments by theses pipelines has been addressed in parts [21], but the authors are, at this point, not aware of studies where these were investigated together. In this work, this became apparent in the differences of the psychrophilic PWASS when analyzed with different pipelines, compared to the thermophilic AD reactors. The algorithm was trained on the (majority of the samples) thermophilic AD reactor communities, where OTU and ASV pipelines produced largely similar results, while results from the two pipelines diverged with the output of the PWASS microbial communities. Such was not only observed in the Permanova and NMDS analysis but could also be seen more directly in the community assembly, where, especially within the PWASS, different taxa or taxa with more differing relative abundances were observed. However, differences in outcome between the pipelines were not limited to the PWASS, and variability in the resulting assignments from the AD reactors, or other samples, might still lead to different conclusions, especially if these are intended to make predictions about the function from taxonomic assignment [62].

## 5. Conclusions

Going forward, while OTUs and clustering pipelines are well established, the use of ASV has the major benefit of direct comparability between studies without the need to reanalyze original data, binding computation and work time and power, while still being accurate down to a single base. ASV algorithms have the capability to even reduce and scale-down computation power, making sequence analysis more accessible on a broad scale. On the flip side, there is still a need on how to actually deal with those from a publication perspective, as there is currently no short-hand available to easily identify them in a written form. Ideas on how to overcome this include using the first few letters of md5 checksums or similar calculation-based short-names, alongside extended supplementary materials, which, however, still do not improve the ease of access from a reader’s perspective. The presented output of this study shows some of the steps that should be considered, as there is a growing need for unification in how to proceed in dealing with the growing mountains of data, with machine learning and AI algorithms and immense possibilities for uncertainties and improvements alike in the interpretation of sequencing data, and the growth of knowledge that comes with them. Furthermore, for comparability, how to better include access to the pipelines and workflows that are currently being used should be explored. This means quality scores or proxies for them, and also more comprehensive ways on which metrics were used to generate data, i.e., common grounds for the settings used in the individual pipelines and based on which criteria they are chosen. Both OTU and ASV approaches can only throw light on “who is potentially present,” but the overall certainty about microbial community composition is based on statistics and algorithms and needs much more than analyzing sequence data.

## Figures and Tables

**Table 1:** Number of taxa derived from the two different sequencing data handling pipelines across different applied abundance and taxa thresholds.

Number of Taxa				
Threshold	OTU Based ^1^	OTU Based ^2^	ASV Based	Merged ^3^
full Dataset	67,015	50,065	8005	
>0.001% relative Abundance	1,204	1,201	1,089	
*Genus* level	910	877	781	1053
*Genus* level >0.001% relative Abundance	285	283	279	348

^1^ = unrarefied; ^2^ = rarefied with vegan; ^3^ = based on rarefied OTU dataset, from merging the datasets, i.e., sum of shared and unique sequences.

**Table 2:** Phyla differences based on choice of pipeline.

Phylum	Treatment	ASV	OTU	∆ of Total Community	Fold Deviation
*Acidobacteriota*	Control	1.12%	<0.01%	1.11%	839
*Armatimonadota*		0.40%	<0.01%	0.40%	510
*Coprothermobacterota*		16.46%	18.45%	2.00%	1.12
*Firmicutes*		21.10%	18.82%	2.28%	1.12
*Hydrothermae*		1.47%	ND	1.47%	∞
*Patescibacteria*		0.41%	0.01%	0.40%	32.6
*Synergistota*		13.62%	15.59%	1.97%	1.14
*Thermotogota*		18.30%	19.48%	1.18%	1.06
*Acidobacteriota*	Experiment	0.64%	<0.01%	0.64%	271
*Armatimonadota*		0.31%	<0.01%	0.31%	519
*Chloroflexota*		0.48%	0.35%	0.13%	1.36
*Coprothermobacterota*		17.44%	18.94%	1.50%	1.09
*Firmicutes*		13.99%	11.91%	2.08%	1.17
*Hydrothermae*		0.71%	ND	0.71%	∞
*Synergistota*		12.90%	14.28%	1.38%	1.11
*Thermotogota*		30.40%	31.43%	1.03%	1.03
*Chloroflexota*	Inoculum	1.24%	0.94%	0.30%	1.32
*Coprothermobacterota*		19.72%	25.31%	5.58%	1.28
*Patescibacteria*		3.22%	0.12%	3.10%	26.9
*Verrucomicrobiota*		0.29%	0.39%	0.09%	1.32
*Acidobacteriota*	PWASS	1.22%	0.29%	0.93%	4.22
*Caldatribacteriota*		0.32%	0.26%	0.07%	1.26
*Caldisericota*		0.43%	0.58%	0.15%	1.36
*Halobacterota*		1.43%	1.83%	0.40%	1.28
*Patescibacteria*		1.00%	0.02%	0.98%	46.2
*Proteobacteria*		34.55%	36.33%	1.78%	1.05
*Synergistota*		2.06%	2.47%	0.41%	1.20

ND = not detected; phyla presented are selected if they are differing by more than 1%, or by a factor <1.2 (20% deviation) if *Phylum* is detected with at least 0.5% relative abundance in one of the two pipelines.

**Table 3:** Simplified Permanova analysis results.

	AD Reactors Only		AD and PWASS	
Explanatory Variable	Without Pipeline Interaction	Including pipeline	Without Pipeline Interaction	Including Pipeline
Pipeline	***	NA	.	NA
Treatment	***	…	***	..
Timepoint	***	…	*	…
Timepoint * Treatment	***	…	*	…
Timepoint * Treatment * Temperature	NA	NA	***	*

Significance codes: NA = not applicable; 0 = ***; 0.001 = **; 0.01 = *; 0.5 = . ; 0.75 = .. ;1 = …

## Data Availability

Sequencing data deposition—All nucleotide sequences obtained in this study were deposited in GenBank. Amplicon sequences from the 16S rRNA gene survey were deposited in the NCBI short read archive with the BioProject ID: PRJNA812418.

## Supplementary Materials

The following supporting information can be downloaded at: https://www.mdpi.com/article/10.3390/bioengineering9040146/s1. Table S1: *Phylum*-level prokaryotic community composition by pipeline and treatment.

## Appendix

**Table A1:** Reactor performance.

	Biogas in mL Day^−1^ Reactor^−1,b^		CH_4_ in % Day^−1^ Reactor^−1^	
Timepoint^c^	Control ^d^	Experiment ^d^	Control	Experiment
1	413.8 ± 18.9	349.6 ± 2.6	69.8 ± 2.8	68.2 ± 3.2
2	476.5 ± 10.9	507.7 ± 15.1	69.8 ± 5.0	69.9 ± 1.2
3	520.0 ± 69.0	587.7 ± 12.0	71.4 ± 6.1	69.2 ± 2.0
4	265.8 ± 88.1	434.6 ± 50.8	73.6 ± 8.8	66.5 ± 2.3
5	367.4 ± 50.1	721.8 ± 11.2	74.3 ± 8.7	70.8 ± 1.9

^a^ not including weekends; ^b^ 500 mL reactor working volume; ^c^ Timepoint equals phase of co-substrate addition to experimental reactors in 10% increments; T1 = 0%–T5 = 40%; ^d^ biogas-yield co-correlating with PWASS-batch quality (VS content).
