# Supplementary material for: Microbiome Analysis via OTU and ASV-Based Pipelines—A Comparative Interpretation of Ecological Data in WWTP Systems
Source: Bioengineering (Basel). 2022 Mar 29;9(4):146. doi: 10.3390/bioengineering9040146 (PMC9029325; doi:10.3390/bioengineering9040146)
Supplement: Supplementary file 1 [file bioengineering-09-00146-s001.zip › bioengineering-1651725-supplementary.pdf]

bioengineering

MDPI

Supplementary Materials

Microbiome Analysis via OTU and ASV-Based Pipelines—A Comparative Interpretation of Ecological Data in WWTP Systems

Jan Torsten Jeske * and Claudia Gallert

Faculty of Technology, Microbiology–Biotechnology, University of Applied Science Emden/Leer, 26723 Emden, Germany; claudia.gallert@hs-emden-leer.de
* Correspondence: torsten.jeske@hs-emden-leer.de

Table S1. Phylum-level prokaryotic community composition by pipeline and treatment.

Phylum	Treatment	ASV	OTU	Δ of total Community
Abditibacteriota	Control	<0.01	<0.01	<0.01
Acidobacteriota		1.12	<0.01	1.12
Actinomycetota		1.13	1.00	0.12
Armatimonadota		0.40	<0.01	0.40
Bacteroidota		7.96	8.09	0.13
Bdellovibrionota		0.01	0.01	<0.01
Caldatribacteriota		1.66	1.65	0.01
Caldisericota		0.09	0.09	<0.01
Campylobacterota		<0.01	<0.01	<0.01
Chloroflexota		0.82	0.71	0.11
Cloacimonadota		3.41	3.47	0.06
Coprothermobacterota		16.46	18.53	2.07
Crenarchaeota		<0.01	<0.01	<0.01
Cyanobacteria		0.04	<0.01	0.04
Dadabacteria		<0.01	<0.01	<0.01
Deferribacterota		<0.01	<0.01	<0.01
Deinococcota		<0.01	<0.01	<0.01
Dependentiae		<0.01	<0.01	<0.01
Desulfobacterota		0.19	0.18	0.01
Dictyoglomota		3.02	3.31	0.29
Elusimicrobiota		<0.01	<0.01	<0.01
Euryarchaeota		2.08	2.16	0.08
FCPU426		<0.01	<0.01	<0.01
Fibrobacterota		<0.01	<0.01	<0.01
Firmicutes		21.05	18.84	2.21
Fusobacteriota		0.03	0.02	<0.01
FW113		<0.01	<0.01	<0.01
Gemmatimonadota		<0.01	<0.01	<0.01
Halobacterota		2.96	3.14	0.18
Hydrogenedentes		0.38	0.38	<0.01
Hydrothermae		1.49	<0.01	1.49
Latescibacterota		<0.01	<0.01	<0.01
LCP-89		<0.01	<0.01	<0.01

Marinimicrobia (SAR406 clade)		<0.01	<0.01	<0.01
MBNT15		<0.01	<0.01	<0.01
Methylomirabilota		<0.01	<0.01	<0.01
Myxococcota		<0.01	<0.01	<0.01
Nitrospirota		0.06	0.05	<0.01
Patescibacteria		0.41	0.01	0.40
Planctomycetota		0.08	0.05	0.02
Proteobacteria		2.38	2.63	0.25
Rs-K70 termite group	Control	<0.01	<0.01	<0.01
SAR324 clade(Marine group B)		0.01	<0.01	0.01
Spirochaetota		0.45	0.43	0.02
Sumerlaeota		0.06	0.02	0.03
Sva0485		<0.01	<0.01	<0.01
Synergistota		13.58	15.50	1.92
Thermoplasmatota		0.03	0.03	<0.01
Thermotogota		18.25	19.47	1.23
Verrucomicrobiota		0.15	0.22	0.07
WPS-2		0.01	<0.01	0.01
WS4		<0.01	<0.01	<0.01
Abditibacteriota	Experiment	<0.01	<0.01	<0.01
Acidobacteriota		1.91	0.01	1.91
Actinomycetota		2.11	1.92	0.19
Armatimonadota		0.94	<0.01	0.94
Bacteroidota		20.77	20.48	0.29
Bdellovibrionota		0.01	0.02	<0.01
Caldatribacteriota		4.41	4.32	0.09
Caldisericota		0.07	0.08	0.01
Campylobacterota		0.01	0.01	<0.01
Chloroflexota		1.41	1.09	0.31
Cloacimonadota		9.66	9.47	0.19
Coprothermobacterota		52.16	56.64	4.48
Crenarchaeota		0.01	<0.01	0.01
Cyanobacteria		0.04	<0.01	0.04
Dadabacteria		<0.01	<0.01	<0.01
Deferribacterota		<0.01	<0.01	<0.01
Deinococcota		<0.01	<0.01	<0.01
Dependentiae		<0.01	<0.01	<0.01
Desulfobacterota		0.23	0.22	0.01
Dictyoglomota		11.39	11.91	0.52
Elusimicrobiota		<0.01	<0.01	<0.01
Euryarchaeota		5.80	5.75	0.05
FCPU426		<0.01	<0.01	<0.01
Fibrobacterota		<0.01	<0.01	<0.01
Firmicutes		41.86	35.78	6.08
Fusobacteriota		0.02	0.02	<0.01
FW113		<0.01	<0.01	<0.01
Gemmatimonadota		<0.01	<0.01	<0.01
Halobacterota		8.13	8.35	0.22
Hydrogenedentes		0.71	0.70	0.01
Hydrothermae		2.14	<0.01	2.14
Latescibacterota		<0.01	<0.01	<0.01

LCP-89	Experiment	<0.01	<0.01	<0.01
Marinimicrobia (SAR406 clade)		<0.01	<0.01	<0.01
MBNT15		<0.01	<0.01	<0.01
Methylomirabilota		<0.01	<0.01	<0.01
Myxococcota		<0.01	<0.01	<0.01
Nitrospirota		0.12	0.12	<0.01
Patescibacteria		0.50	0.02	0.48
Planctomycetota		0.21	0.15	0.06
Proteobacteria		4.07	4.46	0.38
Rs-K70 termite group		<0.01	<0.01	<0.01
SAR324 clade(Marine group B)		<0.01	<0.01	<0.01
Spirochaetota		0.37	0.34	0.03
Sumerlaeota		0.14	0.10	0.04
Sva0485		<0.01	<0.01	<0.01
Synergistota		38.61	42.94	4.32
Thermoplasmatota		0.07	0.05	0.02
Thermotogota		90.96	94.33	3.37
Verrucomicrobiota		0.52	0.73	0.21
WPS-2		0.01	<0.01	0.01
WS4		<0.01	<0.01	<0.01
Abditibacteriota	Inoculum	<0.01	<0.01	<0.01
Acidobacteriota		0.25	0.05	0.20
Actinomycetota		3.08	2.72	0.36
Armatimonadota		0.03	0.04	0.01
Bacteroidota		2<0.01	19.29	0.71
Bdellovibrionota		0.12	0.13	0.01
Caldatribacteriota		1.16	1.12	0.04
Caldisericota		0.03	0.03	<0.01
Campylobacterota		<0.01	<0.01	<0.01
Chloroflexota		1.24	0.95	0.29
Cloacimonadota		<0.01	0.01	0.01
Coprothermobacterota		19.81	25.32	5.51
Crenarchaeota		<0.01	<0.01	<0.01
Cyanobacteria		0.01	<0.01	0.01
Dadabacteria		<0.01	<0.01	<0.01
Deferribacterota		<0.01	<0.01	<0.01
Deinococcota		<0.01	<0.01	<0.01
Dependentiae		0.03	0.02	0.01
Desulfobacterota		0.01	0.02	0.01
Dictyoglomota		0.78	0.75	0.04
Elusimicrobiota		<0.01	<0.01	<0.01
Euryarchaeota		0.55	0.48	0.07
FCPU426		<0.01	<0.01	<0.01
Fibrobacterota	Inoculum	<0.01	<0.01	<0.01
Firmicutes		14.63	14.31	0.33
Fusobacteriota		0.01	0.01	<0.01
FW113		<0.01	<0.01	<0.01
Gemmatimonadota		<0.01	<0.01	<0.01
Halobacterota		0.25	0.20	0.05
Hydrogenedentes		2.39	2.50	0.11
Hydrothermae		0.08	<0.01	0.08

Latescibacterota		<0.01	<0.01	<0.01
LCP-89		<0.01	<0.01	<0.01
Marinimicrobia (SAR406 clade)		<0.01	<0.01	<0.01
MBNT15		<0.01	<0.01	<0.01
Methylomirabilota		<0.01	<0.01	<0.01
Myxococcota		<0.01	<0.01	<0.01
Nitrospirota		0.22	0.20	0.02
Patescibacteria		3.25	0.14	3.10
Planctomycetota		0.10	0.07	0.03
Proteobacteria		7.11	6.08	1.03
Rs-K70 termite group		<0.01	<0.01	<0.01
SAR324 clade(Marine group B)		<0.01	<0.01	<0.01
Spirochaetota		0.04	0.03	0.01
Sumerlaeota		0.29	0.28	0.01
Sva0485		<0.01	<0.01	<0.01
Synergistota		2.58	2.63	0.06
Thermoplasmatota		<0.01	<0.01	<0.01
Thermotogota		21.60	22.22	0.62
Verrucomicrobiota		0.29	0.41	0.12
WPS-2		0.04	<0.01	0.04
WS4		<0.01	<0.01	<0.01
Abditibacteriota	PWASS	<0.01	<0.01	<0.01
Acidobacteriota		1.22	0.29	0.92
Actinomycetota		5.50	5.25	0.25
Armatimonadota		0.02	0.03	0.01
Bacteroidota		8.06	7.96	0.10
Bdellovibrionota		0.25	0.19	0.06
Caldatribacteriota		0.36	0.25	0.11
Caldisericota		0.43	0.59	0.15
Campylobacterota		5.61	5.55	0.06
Chloroflexota		7.16	7.06	0.10
Cloacimonadota		0.26	0.21	0.05
Coprothermobacterota		0.01	0.06	0.05
Crenarchaeota		<0.01	<0.01	<0.01
Cyanobacteria		0.04	0.03	0.01
Dadabacteria	PWASS	<0.01	<0.01	<0.01
Deferribacterota		<0.01	<0.01	<0.01
Deinococcota		<0.01	<0.01	<0.01
Dependentiae		0.03	0.02	<0.01
Desulfobacterota		0.97	1.03	0.06
Dictyoglomota		<0.01	0.01	0.01
Elusimicrobiota		<0.01	<0.01	<0.01
Euryarchaeota		0.07	0.06	0.01
FCPU426		<0.01	<0.01	<0.01
Fibrobacterota		0.01	0.01	<0.01
Firmicutes		24.68	24.90	0.21
Fusobacteriota		3.50	3.33	0.17
FW113		<0.01	<0.01	<0.01
Gemmatimonadota		0.02	0.03	0.01
Halobacterota		1.39	1.80	0.41
Hydrogenedentes		0.44	0.49	0.05

Hydrothermae	<0.01	<0.01	<0.01
Latescibacterota	0.01	<0.01	0.01
LCP-89	<0.01	<0.01	<0.01
Marinimicrobia (SAR406 clade)	0.01	<0.01	0.01
MBNT15	<0.01	<0.01	<0.01
Methylomirabilota	<0.01	0.01	<0.01
Myxococcota	0.13	0.09	0.04
Nitrospirota	0.08	0.09	0.01
Patescibacteria	1.02	0.02	0.99
Planctomycetota	0.17	0.18	<0.01
Proteobacteria	34.55	36.12	1.58
Rs-K70 termite group	<0.01	<0.01	<0.01
SAR324 clade(Marine group B)	0.23	<0.01	0.23
Spirochaetota	0.47	0.48	0.01
Sumerlaeota	0.03	0.02	0.01
Sva0485	<0.01	<0.01	<0.01
Synergistota	2.05	2.48	0.42
Thermoplasmatota	<0.01	<0.01	<0.01
Thermotogota	0.06	0.13	0.07
Verrucomicrobiota	1.06	1.24	0.18
WPS-2	0.09	<0.01	0.09
WS4	<0.01	<0.01	<0.01
